# Measurement of autoantibodies in pediatric- and adolescent-onset systemic lupus erythematosus and their significant relationship with disease-associated manifestations

**DOI:** 10.1186/1546-0096-10-S1-A24

**Published:** 2012-07-13

**Authors:** Brooke E Gilliam, Amanda K Ombrello, Rufus Burlingame, Peri H Pepmueller, Reema H Syed, Terry L Moore

**Affiliations:** 1 INOVA Diagnostics, San Diego, CA, USA; 2 NIH, Bethesda, MD, USA; 3 Saint Louis University, St. Louis, MO, USA; 4 St Louis University HSC, St Louis, MO, USA

## Purpose

Pediatric-onset systemic lupus erythematosus (SLE) patients are prone to develop major organ involvement and a more severe disease course than adult-onset SLE. Early management of the disease is necessary to prevent further complications, which can partly be accomplished by monitoring autoantibody levels and understanding their significance in the disease process. We evaluated an autoantibody profile in pediatric- and adolescent-onset systemic lupus erythematosus (SLE) patients to determine the clinical and statistical associations with disease-related manifestations.

## Methods

Sera from 53 SLE patients and 22 healthy individuals were collected. Antibodies to C1q, histone, chromatin, ribosomal P, double stranded (ds) DNA, and high avidity (HA) dsDNA were measured by enzyme-linked immunosorbent assays. Patient records were evaluated for clinical and laboratory associations.

## Results

SLE patients exhibited significantly elevated levels of all measured autoantibodies when compared to healthy individuals (p<0.05). The most prevalent autoantibody measured in the SLE cohort was anti-C1q antibodies, found in 58% of SLE patients. Anti-C1q antibodies correlated significantly with proteinuria, fever, urinary casts, and decreased complement levels (p<0.05). Anti-C1q antibodies were significantly elevated in SLE patients with active disease (127U) compared to patients who were inactive (63U) (p<0.05). Anti-C1q antibodies and anti-histone antibodies were significantly elevated in patients with class III/IV nephritis compared to class I/II/V nephritis (p<0.05). SLE patients with active nephritis at the time of sample collection demonstrated significantly elevated levels of anti-C1q antibodies compared to those without active nephritis (191U v. 80U, p<0.05), also exhibiting 100% specificity for active nephritis, proteinuria, and urinary casts. Chart-documented anti-dsDNA antibodies were positive in 28 SLE patients, INOVA anti-dsDNA antibodies in 21 patients, and HA anti-dsDNA antibodies in 8 patients. However, measuring HA anti-dsDNA antibodies rather than conventional anti-dsDNA antibodies may prove more accurate by eliminating low avidity, weakly bound antibodies detected by traditional assays. Anti-histone antibodies correlated significantly with leukopenia, hemolytic anemia, and anti-dsDNA and HA anti-dsDNA antibodies (p<0.05).

## Conclusion

These studies indicate measurement of anti-C1q antibodies in pediatric/adolescent-onset SLE patients is important because elevated anti-C1q antibodies are indicative of renal disease activity, showing significant correlation with proteinuria, urinary casts and active nephritis. The HA anti-dsDNA antibody ELISA eliminates potential false-positive results and provides a more reliable and accurate assessment. Overall, antibodies to C1q, histone, dsDNA and HA dsDNA exhibited the strongest association with clinical features, indicating the relevance of measuring all of these antibodies in the pediatric and adolescent SLE population.

## Disclosure

Brooke E. Gilliam: None; Amanda K. Ombrello: None; Rufus Burlingame: Inova Diagnostics, Inc., 3; Peri H. Pepmueller: None; Reema H. Syed: None; Terry L. Moore: None.

